# Comparative Proteomics Reveal the Association between SPANX Proteins and Clinical Outcomes of Artificial Insemination with Donor Sperm

**DOI:** 10.1038/s41598-018-25032-4

**Published:** 2018-05-01

**Authors:** X. M. Wang, Z. Xiang, Y. Fu, H. L. Wu, W. B. Zhu, L. Q. Fan

**Affiliations:** 1 Institute of Reproductive & Stem Cell Engineering, School of Basic Medicine Science, Central South University, Changsha, China; 2 Reproductive & Genetic Hospital of CITIC-Xiangya, Changsha, China; 3 Shenzhen Armed Police Hospital Reproductive Center, Shenzhen, China; 4 Medical center for Human Reproduction, Beijing Chao-yang Hospital affiliated to Capital Medical University, Beijing, China

## Abstract

Semen analysis is used for diagnosing male infertility and evaluating male fertility for more than a century. However, the semen analysis simply represents the population characteristics of sperm. It is not a comprehensive assessment of the male reproductive potential. In this study, 20 semen samples from human sperm bank with distinctive artificial insemination with donor sperm (AID) clinical outcomes were collected and analyzed using a two-dimensional differential in-gel electrophoresis (2D-DIGE); 45 differentially expressed protein spots were obtained, and 26 proteins were identified. Most differentially expressed proteins were related to sperm motility, energy consumption, and structure. These identified proteins included several sperm proteins associated with the nucleus on the X chromosome (SPANX) proteins. This prospective study aimed to investigate the association between the expression levels of SPANX proteins and the AID clinical outcomes. The proteins identified in this study provided a reference for the molecular mechanism of sperm fertility and revealed a predictive value of the SPANX proteins.

## Introduction

Over the past century, the diagnosis and treatment of male infertility have been significantly based on semen analysis, which has been used as an integral part of routine clinical infertility practice since the 1940s^1^. However, patients cannot be considered fertile based only on the normal semen analysis. It was shown that 30% of patients with normal semen analysis were associated with abnormal sperm function^2^. The uncertainty affects the efficacy and safety of the treatment. Until recently, the overall pregnancy rate per cycle remains to be 12.46–14.4% for intrauterine insemination treatment (IUI). Besides, clinical and laboratory evidence indicates that male fertility is not always apparent with semen analysis^3,4^. Therefore, other examination methods, including sperm function tests such as sperm DNA fragmentation/oxidative stress, sperm–zona pellucida binding/*in vitro* penetration tests, and acrosome reaction tests, are used^5^. Still, these tests have not achieved satisfactory advances as clinical routine practices.

The standard semen analysis is symptomatic diagnosis and reflects the population characteristics of sperm seen under the microscope. Human spermatogenesis involves thousands of genes and subtle reactions; any mistakes can result in infertility or subfertility^6–8^. Semen analysis simply displays the behavior of sperm cells. The underlying cause of male infertility or subfertility has not been fully revealed yet. The intact structure and function of sperm largely depend on active proteins. Thus, the proteomics analysis is able to provide new insights into sperm dysfunction and male fertility^9,10^. In the Human Sperm Bank in Reproductive and Genetic Hospital of CITIC-Xiangya, the sperms of few donors failed to conceive any pregnancy after 12 inseminations with donor sperm (AID) treatment cycles with different recipients (pregnancy rate, PR = 0, low-fecundity group). On the contrary, within 5–10 AID treatment cycles, the sperms of some donors reached the upper limit of pregnancies (5 pregnancies in China, PR ≥ 50%, high-fecundity group). The semen sample of 10 donors in each group were randomly chosen for further two-dimensional differential in-gel electrophoresis (2D-DIGE). A group of differentially regulated proteins was identified, and subsequent bioinformatics analyses indicated that these proteins were essential to a variety of cellular processes and structures, including spermatogenesis, cell signaling, cell skeleton, and metabolism. Among these identified proteins, several sperm proteins associated with the nucleus on the X chromosome (SPANX) proteins drew the attention of the researchers of this study. In a previous study, SPANX proteins were also identified to be down-regulated in globozoospermia sperm using a DIGE–mass spectroscopy approach^11^. Therefore, this study aimed to explore the expression of SPANX proteins in sperm cells and the predicted value of SPANX proteins on AID. This study helped enhance the understanding of male fertility regulation, guiding the clinical diagnosis and treatment of male infertility.

## Results

### DIGE analysis and MALDI-TOF MS identification

**Figure 1:**
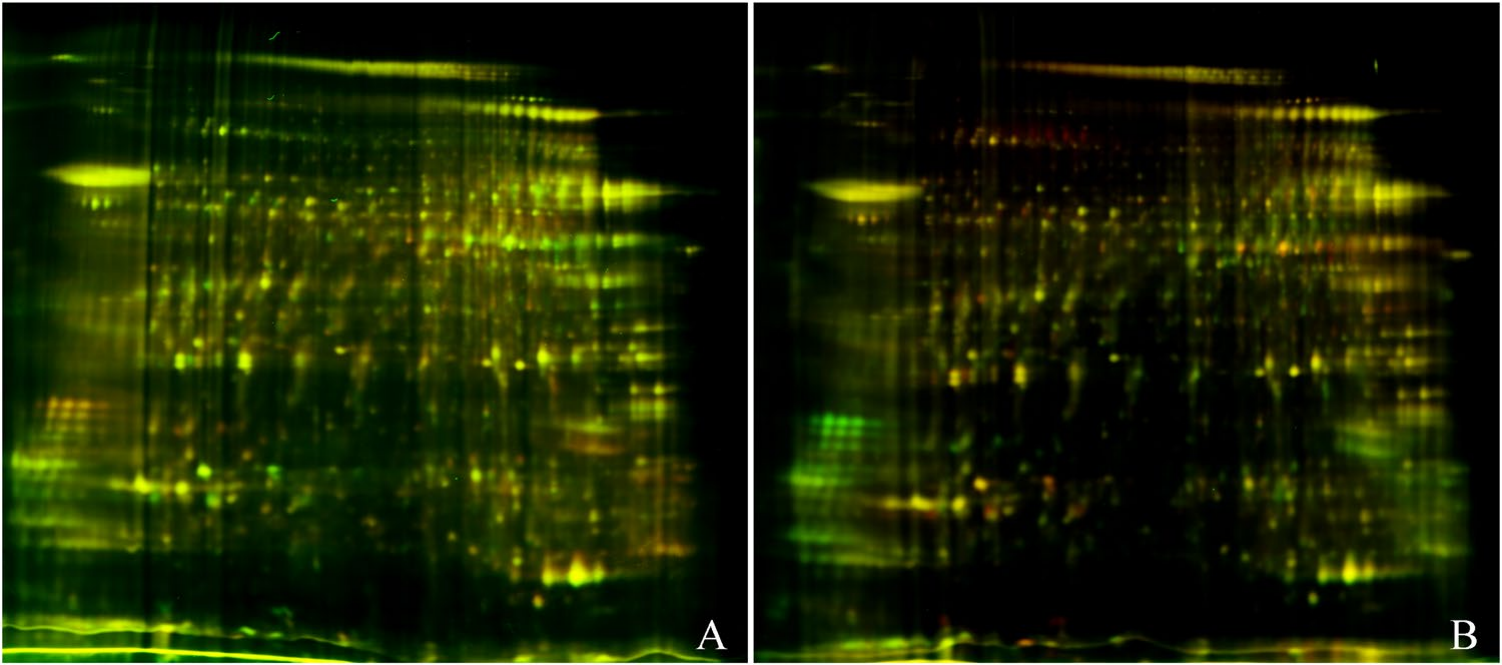
Representative merged 2D-DIGE gels images form sperm proteins of low- and high- fecundity group. Figure A represents the overlapped image of low- and high- fecundity group, which were labeled with cy5 and cy3 respectively; the CyDyes labels swapped in Figure B. (Cy3 = green color, Cy5 = red color, scanned by typhoon 9410).

**Figure 2:**
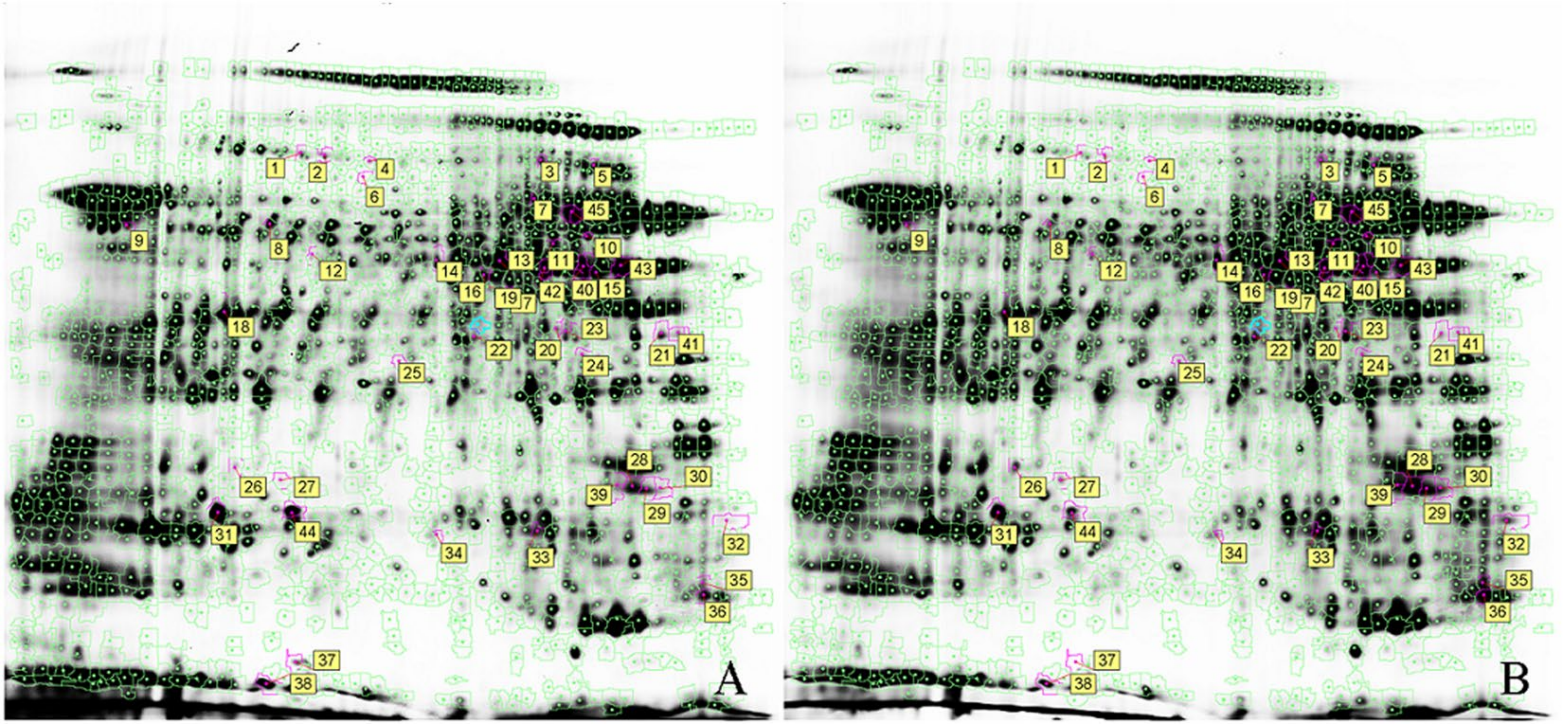
The distribution of differential expression proteins. Figure A&B shown the distribution of differential proteins in high-fecundity group and low-fecundity group separately. Pink cycles shown the protein spots with statistically significance. Analyzed by DeCyder Differential Analysis Software (paired t-test, p < 0.05).

**Figure 3:**
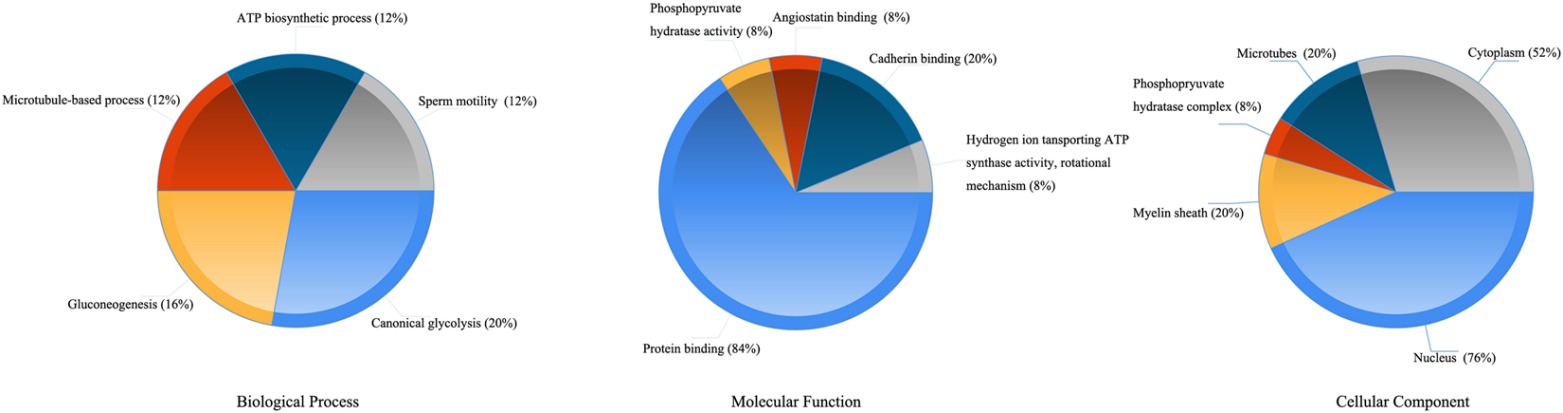
Gene Ontology analysis of the identified proteins. The top 5 biological process, molecular function and cellular component were shown in pie chart.

In gel 1, a 50-μg sperm protein extract of a high-fecundity group was labeled with Cy3, whereas a 50-μg extract of a low-fecundity group was labeled with Cy5. To ensure the reproducibility and reliability, in gel 2, the labeled CyDyes were swapped. Both gels with a Cy2-labeled sample contained an equal mixture of all samples as an internal standard. After 2D gel electrophoresis, the Cy2, Cy3, and Cy5 channels were individually imaged from the gels using mutually exclusive excitation and emission wavelengths (Fig. 1). As shown in Fig. 2, different spots between the two groups were obtained, with the pink cycles showing the protein spots with statistical significance. In total, 45 statistically significant protein spots were detected in this study (paired *t* test, *p* < 0.05, Cy3/Cy2 ratio). Of these spots, 21 spots were up-regulated in the low-fecundity group, whereas 24 spots were down-regulated. When the volume–ratio threshold was set at threefold, only 35 (3.4%) of the spots showed differential expression between the two groups. Targeted proteins were excised from the gel and digested with trypsin. MALDI-TOF/MS was applied subsequently and 26 proteins were identified (Table 1). Most of the identified proteins were related with energy consumption, motility, and cell skeleton (Fig. 3).

**Table 1:** The differential proteins identified by MALDI-TOF/MS.

	Identified Protein	SYMBLE	SWISSPROT ID	Mr(Da)	Score	Up or Down in low fertility sperm
1	Outer dense fiber protein 2	ODF2	Q5BJF6	73292.6	472	Down
2	A-kinase anchor protein 4	AKAP4	Q5JQC9	93385.1	385	Down
3	Pyruvate kinase	PKM	P14618	65888.8	245	Down
4	T-complex protein 1 subunit gamma	CCT3	P49368	60424.3	80	UP
5	Glyceraldehyde-3-phosphate dehydrogenase, testis-specific	GAPDHS	O14556	44472.8	178	Down
7	ATP synthase subunit beta, mitochondrial	ATP5B	P06576	56524.6	590	Down
8	ATP synthase subunit alpha, mitochondrial	ATP5A1	P25705	59713.6	227	Down
9	Alpha-enolase	ENO1	P06733	47139.3	326	Down
10	Elongation factor 1-gamma	EEF1G	P26641	56114.4	235	UP
11	Fructose-bisphosphate aldolase A	ALDOA	P04075	39395.3	140	Down
12	Tubulin alpha-3C/D chain	TUBA3C	Q13748	49927.6	177	Down
13	Tubulin alpha-3EChain	TUBA3E	Q6PEY2	49884.6	177	Down
14	L-lactate dehydrogenase A-like 6B	LDHAL6B	Q9BYZ2	41916.2	89	Down
15	SRA stem-loop-interacting RNA-binding protein,mitochondrial	SLIRP	Q9GZT3	12341.4	59	UP
16	Mirror-image polydactyly gene 1 protein	MIPOL1	Q8TD10	30255.6	43	UP
17	Uncharacterized protein C22orf31	C22orf31	O95567	32634.3	40	UP
18	E3 ubiquitin-protein ligase RNF34	RNF34	Q969K3	41613.5	46	UP
19	ADP-ribosylation factor-related protein 1	ARFRP1	Q13795	22599.4	34	UP
20	Tubulin beta-6 chain	TUBB6	Q9BUF5	50058.1	76	Down
21	Peroxiredoxin-6	PRDX6	P30041	25019.2	113	Up
22	Sperm protein associated with the nucleus on the X chromosome B1	SPANXB1	Q9NS25	11832.8	127	Down
23	Sperm protein associated with the nucleus on the X chromosome A1/A2	SPANXA1	Q9NS26	11790.7	104	Down
24	Sperm protein associated with the nucleus on the X chromosome C	SPANXC	Q9NY87	10995.4	107	Down
25	Sperm protein associated with the nucleus on the X chromosome D	SPANXD	Q9BXN6	11022.4	360	Down
26	Gamma-enolase	ENO2	P09104	47239	171	Down

### Western blotting of SPANX-A/D proteins

**Figure 4:**
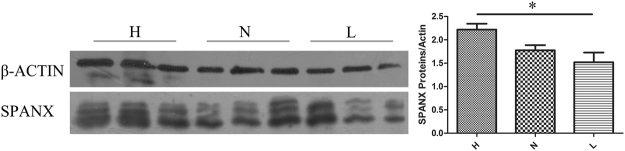
The western blotting detected SPANX proteins expression in high fecundity group (H, pregnant rate > 50%), normal group (N, pregnant rate = 25%), low fecundity group (L, pregnant rate = 0), Figure B revealed the expression levels between the three group (gray value ratio of target protein and β-actin, paired t test, *p < 0.05.

The SPANX-A/D subfamily encodes several protein ranges in 15–20 kDa with high similarity. Thus, the primary antibody (sc-162262, Santa Cruz, Dallas, USA) was unable to separate the proteins distantly. The expression levels of SPANX proteins were evaluated using the gray value of the target protein/reference protein. The target proteins expression in the low-fecundity group was reduced compared with that in the high-fecundity group significantly (Fig. 4, paired *t* test, *p* < 0.05).

### Prospective study

Based on the expression level of the SPANX proteins detected by WB, K-mean clustering analysis was performed and verified using the independent-sample *t* test (*p* = 0.041 < 0.05). According to the cluster analysis, the samples were divided into two groups, as shown in the Table 2, the high-expression group (*n* = 18) and low-expression group (*n* = 88). The age, endometrial thickness, and BMI of female recipients fit the normal distribution between the two groups. No statistically significant differences were found. Moreover, the semen parameters used for the treatment (concentration, motility both before and after cryopreservation) were not statistically significantly different either. The chi-square test was conducted to compare the clinical pregnancies between the two groups; the clinical pregnancies of the high-expression group were significantly more than those of the low-expression group (Table 2, p = 0.005 < 0.01).

**Table 2:** Parameters of female recipients and routine parameters of semen sample applied for treatment between the low- and high- expression groups.

	Low expression group (n = 88)	High expression group (n = 18)	p
Expression level	0.87 ± 0.18	1.08 ± 0.16	0.041
Recipients age	28.69 ± 3.56	30.44 ± 3.51	0.773
Endometrial thickness(mm)	10.76 ± 1.57	10.07 ± 1.27	0.195
BMI(kg/m2)	21.57 ± 1.66	21.08 ± 1.81	0.593
Donors age	21.21 ± 2.93	21.52 ± 2.21	0.425
Concentration (×106/ml)			
Before cyropreservation	64.8279 ± 7.52	67.69 ± 7.39	0.253
After cyropreservation	53.69 ± 3.66	52.44 ± 2.46	0.112
Motility (%)			
Before cyropreservation	51.15 ± 2.64	51.73 ± 3.21	0.423
After cyropreservation	42.5 ± 2.46	41.8 ± 3.29	0.169
Pregnant rate	17.05%	50%	0.005

## Discussion

Male fertility can be defined as the successful fertilization of oocytes by sperm; the resulting zygotes undergo embryonic and fetal development until birth^12^. In clinical practice, the semen analysis, one of the major cornerstones in evaluating fertility, is routinely used for diagnosing male infertility. It is an essential reference for doctors to judge the male fertility status. Semen analysis mainly focuses on the concentration, motility, and sperm morphology, and includes semen volume, pH, liquefaction time, and so forth. However, human spermatogenesis involves thousands of genes and subtle reactions; any mistake can result in infertility or subfertility. In many cases, patients with normozoospermic still undergo infertility^4,13^. The semen parameters of the two groups collected from the human sperm bank were far beyond the normal standard in the present study. Yet, the AID clinical outcomes were distinctive. The low-fecundity group did not mean infertility, but different recipients did not have successful pregnancy by artificial insemination.

According to the reports of the WHO, only the female factor causing infertility accounts for about 70% of the infertility couples, while the proportion of male factor accounts for about 50%; 20–30% of the infertility is due to both female and male factors. Therefore, in the monogamy situation, male fertility cannot be sufficiently predicted. The sperm in human sperm bank, due to different female recipients may judge the male/sperm factor more definitely to a certain degree.

The standard semen analysis provides detailed information about the population parameters of sperm but lacks assessment of a single sperm cell. Along with the development of assisted reproductive techniques (ART), especially the clinical application of intracytoplasmic sperm injection (ICSI), the demand for objectively and accurately assessing sperm at a single-cell level is increasing. The standard semen analysis, which is performed routinely in clinical practice, is unable to distinguish the infertile patients from the fertile ones faultlessly^4,13^. The analysis results only reflect the semen status for patients. This indicates that semen parameters only, as diagnostic criteria, are inadequate. Other test methods need to be developed to thoroughly characterize male infertility.

The underlying reason of male infertility or subfertility, which is probably caused by any mistakes in the complicated spermatogenesis and subtle reactions, has not been fully revealed. These unrevealed bioactivities become a stumbling block in developing new examination methods. The study of potential fertility biomarkers provided a new insight for measuring male fertility. Because of the complicated spermatogenesis and the unique sperm structure, the protein markers seem to be the optimal choice for clinical diagnostic and prognostic markers. Vitality and sperm major functions are mostly achieved through proteins. Moreover, human sperms are highly specialized cells. The sperm cells are unable to transcript or translate, and all proteins required for reproduction are synthesized during spermatogenesis. This makes proteins good research objects of fertility^14^. The human sperm cell is well suited for proteomic studies because it is accessible, can be easily purified, and is believed to be transcriptionally silent^15^. Based on these considerations, the 2D DIGE was performed to analyze the proteomics difference between the low- and high-fecundity groups. In this study, 26 differentially expressed proteins were identified. Most of these proteins were related to cell movement, energy consumption, and cell skeleton.

Since artificial insemination is different from *in vitro* fertilization, the motility function of sperm is still largely needed to exercise until the sperm–ovum fusion. The proteomics results in this study revealed many proteins related to sperm motility, which were also down-regulated in the low-fecundity group. A-kinase anchoring proteins were the main flagella proteins. These proteins regulated the human sperm flagella by promoting phosphorylation. Outer dense fiber proteins were specifically expressed in sperm flagella and were essential components of flagella^16,17^. Moreover, the energy supply was a prerequisite for the exercise of motility. Testis-specific glyceraldehyde-3-phosphate dehydrogenase glyceraldehyde-3-phoshoate (GAPDHS) was one of the key enzymes in the glycolysis pathway, and it mainly catalyzed the conversion of glyceraldehyde-3-phosphoric acid into glycerate-3-phosphate^18^. The GAPDHS knockout mice produced nonmotile sperm^19,20^. Fructose diphosphate aldolase A (ALDOA) catalyzed the conversion of fructose-1, 6-diphosphate into glyceraldehyde-3-phosphate and dihydroxyacetone phosphate. The lack of ALDOA blocked the glycolysis^21^. ATP5B and ATP5A1 were the subunits of ATP synthase. All the aforementioned proteins were closely related to the energy consumption and ATP synthesis, and also down-regulated in the low-fecundity group, indicating that the sperms of the low-fecundity group underwent undetectable changes in energy metabolism pathways. Both enolase 1&2 (ENO1&2) are important glycolytic enzymes and played a key role in aerobic glycolysis^22^. These energy consumption–related proteins were down-regulated in the low-fecundity group. In consideration of the samples collected from human sperm bank with perfect semen parameters, these protein changes somehow subtly influenced the sperm function, which remains unrevealed.

Some of the abnormally expressed proteins also remain unknown functions, such as the SPANX proteins. They were found to be down-regulated in the low-fecundity group. The SPANX multigene family includes two subfamilies, SPANX-A/D and SPANX-N^23^. The SPANX genes, which evolved rapidly in primates, are located on the X chromosome and reside in the Xq26.3–Xq27.3 region. The SPANX gene family encodes several 15 to 20-kDa proteins. It is restricted to the normal testis and certain tumors^24–26^. The interest in the SPANX genes is largely because they are also specifically expressed as cancer/testis-specific antigens in tumors, making them conceivable candidates for cancer immunotherapy^25,27,28^. In human spermatozoa, some SPANX proteins are found in the spermatozoa carters, the presence of which is controversially linked to reduced fertility in mammals. The SPANX-A/D proteins were first detected in the nuclear envelope of early round spermatids in the Golgi phase of acrosome biogenesis. The SPANX-A/D proteins migrate as a distinct postacrosomal domain of the nuclear envelope toward the base of the nucleus as nuclear condensation and elongation proceed. In the mature spermatids, SPANX-A/D proteins then associate with the redundant nuclear envelope within the residual cytoplasm. The localization of SPANX-A/D to a subpopulation of spermatids and spermatozoa suggested the precise temporal and spatial distribution of SPANX-A/D proteins in postmeiotic spermatid nuclei^29^. However, the exact physiological function of the SPANX protein family remained unclear. Interestingly, the SPANX-A/D proteins were also down-regulated in globozoospermia in a previous study. The expression of SPANX-A/D proteins between the low- and high-fecundity groups was significant. Moreover, the individual variation in SPANX-A/D proteins was observed between the low-fecundity and normal groups. The variation indicated that the SPANX-A/D proteins might undergo unknown function connected with fertility. In several proteomics studies of human sperm, the SPANX-A/D proteins were also found to relate to oxidative stress, asthenozoospermia, and normozoospermic patients with infertility.

Luna Samanta *et al*. found the SPANX proteins were the target proteins of SUMO1, SUMO1 is one of the major isoforms of mammalian SUMO proteins, involving SUMOylation, which is a post-translational modification (PTM), indication that the SPANX proteins may experience SUMOylation modification after translation^30^. SUMO1 is confirmed to be associated with sperm motility, morphology and DNA damage^31^. Interestingly, several researchers have identified that SPANX proteins were related with sperm motility, morphology and DNA damage. In asthenozoospermia patients, several SPANX proteins were found to be down regulated in asthenozoospermia sperm^32–34^. In our previous study of globozoospermia proteomics, the SPANX proteins also found to be down regulated^11^. Intasqui found that the SPANX proteins were also related to the DNA fragmentation^35^. These coincidences indicated that the SPANX proteins may relate to the SUMOylation. SPANX proteins were also found related to the reactive oxygen species (ROS) by a proteomic approach^36^, the role of ROS in sperm has been widely discussed, and been considered associated with male fertility, such as DNA damaged, motility, the relationship between ROS and SUMOl tion in human sperm are not fully described and researched, but the ROS/SUMO axis was reported to contribute to the response of acute myeloid leukemia cells to chemotherapeutic drugs^37^. The findings listed above raised a speculation, a possible biological process which SPANX proteins may involve, that the ROS produced by stress or abnormal condition affected the SUMOlyzation of SPANX protein/proteins, which led to the increase of sperm DNA damage, and finally affecting the sperm fertility. Currently, our group is working on confirming our hypothesis, starting with the relationship of SPANX protein and sperm motility.

Thus, this prospective study was performed to elucidate the correlation between the expression level of SPANX proteins and the AID clinical outcomes. The clinical pregnancies of the high-expression group were significantly more than those of the low-expression group (*P* = 0.005 < 0.01). Although the preliminary experimental results revealed that the expression level of SPANX proteins was related to the AID clinical outcomes, several conditions needed to be optimized. For instance, the antibody used could not efficiently distinguish the specific member of the family due to high similarity. Hence, antibodies still need to be developed to expatiate the detailed relationship between the SPANX proteins and human fertility. Nevertheless, the overall expression level of the SPANX family still indicated the relationship pattern. The exact member/s of SPANX proteins and mechanisms involved in male fertility should be the key areas of future investigation.

In summary, the 26 proteins identified in this study provided a reference for the molecular mechanism of sperm fertility. The sperm cell structure and energy consumption may be one of the reasons of decreasing fertility, but the fertilization is never determined by one or several factors. The whole process is highly complicated and far beyond human understanding. Similarly, the human SPANX proteins also potentially exercise unknown functions affecting male fertility. A prospective research is not enough to fully prove the SPANX proteins as a fertility marker. However, it still points out the next main emphasis.

## Methods

### Ethical statement

According to the *Basic Standards and Technical Specification of Human Sperm Bank*, *Ethical Principle of Human Sperm Bank* and *Regulation of Human Sperm Bank* issued by National Health and Family Planning Commission of the People’s Republic of China, both personal information of sperm donors and recipients must be confidential, thus no information that could identify participants in this article can be found.

This study was carried out with the approval and under the supervision of Ethic Committee of Basic Medical Science School, Central South University (Approve No.: LLSB-2017-002). All the semen samples used for 2D-DIGE and Western blotting analysis were obtained from each donor with informed consent signed in their first donation. Additionally, for the prospective study, the trace amount of semen samples collected prior to the AID treatment were also collected with informed consent signed by recipients couples which also contained the approval of case information collection. All experiments and procedures were reviewed and approved by the Ethic Committee of Basic Medical Science School, Central South University, and were performed in accordance with the relevant guidelines and regulations.

### Sample collection

For proteomics analysis, 20 semen samples from the low- and high-fecundity groups were randomly chosen from healthy donors enrolled at the Human Sperm Bank in Reproductive and Genetic Hospital of CITIC-Xiangya. Nine semen samples were prepared (three samples each were randomly chosen from the low- and high-fecundity groups and three samples were randomly chosen with an average clinical pregnancy rate) for the Western blot verification of identification results. All the collected semen samples met the requirements of the fifth edition manual of the World Health Organization (WHO).

### Prospective study

A total of 106 couples receiving AID treatment were recruited in this prospective study from October 2014 to March 2016. The exclusion criteria: recipients with medical history of endometriosis, Stein-Leventhal syndrome, endometrial polyp, pelvic adhesion, as well as the recipients receiving therapy for inducing ovulation. The inclusive criteria: recipients with age under 35-year-old, the thickness of endometrial >8 mm, no adverse pregnancy history, at least one side of the fallopian tube was smooth. All the donor semen applied for the treatment fit the requirements of *the Basic Standards and Technical Specification of Human Sperm Bank*, the trace semen sample (50 µL) was collected immediately after wash but prior to implementing the treatment. The expression level of SPANX proteins was evaluated by Western blot. The treatment group was divided into two subgroups based on the expression values.

### Spermatozoa isolation and protein extraction

The spermatozoa cells were isolated from the semen using a two-step percoll gradient (Sigma-Aldrich, Cat. # F0119; 45% and 90%, diluted in Ham’s F10, HyClone, Cat. # SH30025.01) and centrifuging at 600 g for 30 min. The spermatozoa pellet was collected and washed with phosphate-buffered saline thrice by centrifuging at 600 g for 10 min at room temperature. The spermatozoa were pooled, frozen immediately after the last centrifugation, and stored in liquid nitrogen until use. For both the DIGE and Western blot procedures, the frozen samples were thawed at 37 °C after centrifugation at 600 g for 10 min at 4 °C, and the resulting supernatants were discarded. The purified spermatozoa were solubilized in lysis buffer on ice. The suspension was treated using the ultrasonic instrument. After ultrasonic treatment, the samples were put on ice for 45–60 min. After centrifugation, all non-solubilized material was removed. The protein concentration was determined with a Bradford assay kit (Bio-Rad, Cat. #5000006) using albumin diluted in lysis buffer as the reference standard. The supernatant was stored at −80 °C for future use.

### Protein labeling with CyDye DIGE Fluor

The sample labeling process was carried out according to the Ettan DIGE user manual. A total of 50 µg protein sample was labeled with 400 pmol of Cy3 or Cy5. An internal reference standard, consisting of two mixed samples, was used in the experiment and labeled with Cy2. The labeling reaction was conducted on ice for 30 min under dark conditions. Reactions were quenched by adding 10 mM lysine and incubated for 10 min on ice under dark conditions. The labeled samples were pooled for the subsequent steps in the experiment. Each sample was separated twice on separate gels to demonstrate the reproducibility and reliability of the results of the present study.

### Protein separation by 2D fluorescence DIGE

A 2D electrophoresis was performed as described in a previous study with several minor modifications. Immobilized pH gradient (IPG) strips were hydrated in hydration buffer and incubated with labeled samples under dark conditions. The first-dimension isoelectric focusing (IEF) was performed using an Ettan IPGphor System (GE Healthcare, Pittsburgh, USA) for a total of 60 kVh at 20 °C, specifically in five steps: 30 V for 12 h, 120 V for 1 h, 500 V for 1 h, 1000 V for 1 h, and 8000 V for 7 h. After the IEF, the IPG strips were equilibrated in the equilibration solution containing 1% w/v dithiothreitol (DTT) for 15 min and then in the same solution containing 2.5% IAA instead of DTT. The strips were then subjected to 2D electrophoresis after transferring them onto 12.5% sodium dodecyl sulfate–polyacrylamide gels. Electrophoresis was performed at 2.5 W per gel for 30 min, followed by 20 W per gel until the bromophenol blue reached the end of the gel.

### Scanning of DIGE-labeled images and image analysis

Protein spots in the analytical gels were visualized by Coomassie brilliant blue G-250 staining. Labeled proteins were visualized using the Typhoon 9410 imager (GE Amersham Biosciences, Pittsburgh, USA). The Cy5/Cy3/Cy2 images were scanned. All gels were scanned at 100 µm resolution. The stained gels were scanned using the UMax Powerlook 2110XL (UMax, Dallas, USA), and the image analysis was performed using the ImageMaster 2D Platinum version 5.0 (GE Amersham Biosciences, Pittsburgh, USA). The images were analyzed using the DeCyder Differential Analysis Software (GE Amersham Biosciences, Pittsburgh, USA). Differential in-gel analysis (DIA) was applied to calculate protein abundance variations between samples on the same gel. The resulting spot maps were analyzed through biological variation analysis to provide statistical data on the differential protein expression that existed between the two groups. The Cy3/Cy2 and Cy5/Cy2 DIA ratios were used to calculate the average protein abundance changes. The benefit of the internal reference standard was that it increased the investigator’s confidence in results obtained from different gels.

### Spot picking and enzymatic digestion

Separate preparative gels were run to obtain sufficient amounts of protein for MS analysis. In brief, gel pieces were first stained and dried in a vacuum pump. Then, the spots were dehydrated, digested using trypsin digestion working solution, and incubated for 15–18 h. Supernatants were collected and vacuum-dried. The extraction was performed in the following steps. (1) The supernatants were dissolved in 50% acetonitrile (ACN) and 0.5% trifluoroacetic acid (TFA) in a 37 °C water bath for 60 min and ultrasonically processed for 15 min. (2) 75% ACN and 0.5% TFA were further added in a 37 °C water bath for 60 min and ultrasonically processed for 15 min. (3) 100% ACN and 0.5% TFA were again added in a 37 °C water bath for 60 min and ultrasonically processed for 15 min.

### MALDI analysis

Peptide profiles were analyzed with an ultraflex TOF/TOF (Bruker Daltonics, Massachusetts,USA). The samples were desalinated and concentrated using ZIP-TIPTM (Millipore, Perkin Elmer, UK) and then mixed with a-cyano-4-hydroxycinnamic acid matrix solution according to the proportion of 1:1. The mixture was deposited in the AnchorChip (Bruker Daltonics, Massachusetts,USA) for future use. The reflex pattern was automatically controlled by the FlexControl software. The Mascot software (Matrix Science Ltd, London, UK) was used to analyze the PMF and LIFT MS peak. The BioTools software (Bruker Daltonics, Massachusetts,USA) was used to search and identify PMF and MS/MS data with a peptide mass tolerance of 100 ppm. Protein identifications were accepted when the observed and predicted isoelectric points and relative molecular weights were consistent, and scores indicated nonrandom identifications at a significance level of P < 0.05.

### Western blotting

Protein exacts and prestained protein standards (Thermo Fisher, Cat. # 26616) were separated by SDS-PAGE. The conventional precast polyacrylamide gels with a 4–15% gradient were applied for the protein electrophoresis (Bio-Rad, Cat. # 5678081). The precast gels were run for 45 min at constant voltage 200 V in running buffer (25 mM Tris base, 192 mM glycine, 1% SDS, pH 8.3, as recommended by the manufacturer). While the electrophoresis was finished, the proteins were transferred to a polyvinylidene difluoride membrane (Millipore, Cat. # IPVH00010) using the Bio-Rad mini trans-blot electrophoretic transfer cell at constant current 200 mA for 60 min. The electrophoresis procedure was carried out at room temperature, the membrane transfer procedure was carried out on ice. Then the membranes were blocked with 5% skim milk (Thermo Fisher, Cat. # LP0031B) in TBST (20 mM Tris, 150 mM NaCl, containing 0.05% Tween-20, pH 7.4) at room temperature for 2 h, then incubated with primary antibodies. The concertation of primary antibodies was modified according to the product description, the target protein antibody was anti-SPANX A/D antibody (Santa Cruz, sc-162262, 1/500, diluted in TBST), the control protein antibody was anti-β-Actin (Abcam, ab8227,1/2000 diluted in TBST). The primary antibodies were incubated at 4 °C overnight (approximately 15 h). The primary antibody was removed and washed in TBST thrice for 5 min each. The secondary antibody (goat anti-rabbit IgG H&L, Abcam, Cat. # ab205718) was diluted 1/2500 with TBST then incubated with membrane at room temperature for 1 h, followed by membrane wash thrice for 5 min each with TBST. Then the membranes were exposed to clarity enhanced chemiluminescence (ECL) reagent (GE healthcare, Cat. # RPN2133) in the dark. The film (Carestream, Cat. # 864 6770) was exposed in the dark for 2–3 min at room temperature, then the film was developed for 5 min in the develop solution (Carestream, Cat. # 526 9055) and fixed for 25 min in the fix solution (Carestream, Cat. # 886 8804), and washed thrice in filtered water, the dry film was scanned and analyzed. The expression level was represented by the gray value ratio of target protein and β-actin.

### Statistical methods

The Cy3/Cy2 and Cy5/Cy2 DIA ratios, SPANX proteins expression levels of low- and high-fecundity groups and normal group were statistically analyzed by paired t test. The SPANX proteins expression levels of prospective study was clustered by direct clustering method, and independent sample t test was used to evaluated the classification. The chi-square test was used to analyze the correlation between the expression of SPANX protein and the clinical pregnancies of AID.

### Bioinformatics analysis

Gene Ontology analysis was performed by FunRich 3.0 (Functional Enrichment analysis tool, http://www.funrich.org/).
